# The relative risk of clinically relevant cholelithiasis among glucagon-like peptide-1 receptor agonists in patients with type 2 diabetes mellitus, real-world study

**DOI:** 10.1186/s13098-024-01526-2

**Published:** 2024-12-04

**Authors:** Mohammed Ali Gameil, Elshahat Ali Ahmed Mohamed Yousef, Rehab Elsayed Marzouk, Mohamed H. Emara, Abeer H. Abdelkader, Rasha Ibrahim Salama

**Affiliations:** 1 Endocrinology Unit, Internal Medicine Department, Faculty of Medicine, Mansoura University, Mansoura, Dakahlia Egypt; 2 Nephrology Unit, Internal Medicine Department, Faculty of Medicine, Mansoura University, Mansoura, Dakahlia Egypt; 3 Medical Biochemistry and Molecular Biology Department, Faculty of Medicine, Helwan University, Helwan, Cairo, Egypt; 4 Hepatology, Gastroenterology, Infectious Diseases Department, Faculty of Medicine, Kafrelsheikh University, Kafrelsheikh, Egypt; 5 Department of Internal Medicine, Alyousif Hospital, Alkhobar, Kingdom of Saudi Arabia; 6 Tropical Medicine Department, Faculty of Medicine, Zagazig University, Zagazig, Alsharqia Egypt

**Keywords:** GLP1-receptor agonists, Relevant, Biliary, Cholelithiasis, Type 2 diabetes mellitus

## Abstract

**Background and Aim:**

The association between biliary disorders with weight reduction enhanced by GLP-1RAs was observed frequently, nevertheless, the relative risk of the clinically relevant cholelithiasis was not specified clearly among different GLP-1RAs.

**Methods:**

308 patients with type 2 diabetes mellitus (T2D) were recruited and divided into 4 groups; liraglutide, dulaglutide, semaglutide, versus control group; comprised of 69, 76, 71, and 92, respectively. Clinical history, examination, laboratory, and radiology tests were implemented.

**Results:**

Cholelithiasis significantly associates GLP1-RAs (p = 0.033). Overall cholelithiasis was evident in 31.2% of our participants. Symptomatic cholelithiasis prevails in 60.4% of patients with cholelithiasis. Symptomatic complicated cholelithiasis prevailed in 33.3%; distributed in 28.1%, 28.1%, 21.9%, and 21.9% in liraglutide, semaglutide, dulaglutide, and control groups, respectively. Meanwhile, symptomatic uncomplicated cholelithiasis was observed in 27.1%; distributed in 34.6%, 30.8%, 15.4%, and 19.2% in Liraglutide, semaglutide, dulaglutide, and control groups, respectively. Asymptomatic cholelithiasis was noted in 36.8%, 21.1%, 10.5%, and 31.6% of patients with dulaglutide, semaglutide, liraglutide, and control groups, respectively. Specifically, 81.1%, 68%, and 44% of patients with liraglutide, semaglutide, and dulaglutide experienced symptomatic cholelithiasis. The relative risk of cholelithiasis was 1.2, 1.3, and 1.4 in liraglutide, dulaglutide, and semaglutide with number needed to harm of 17.25, 14.69, and 10.96, respectively. The relative risk of symptomatic cholelithiasis was 1.6, 0.9, and 1.4 in liraglutide, dulaglutide, and semaglutide with number needed to harm of 3.14, 16.67, and 5.56, respectively.

**Conclusion:**

Liraglutide was associated with the highest risk of clinically relevant cholelithiasis than semaglutide, and dulaglutide in patients with T2D.

## Background

Glucagon-like peptide-1 receptor agonists (GLP-1RAs) are incretin-based agents, used for treatment of type 2 diabetes (T2D) and obesity. It mimics the endogenous hormone glucagon‐like peptide-1 (GLP‐1). GLP‐1 is a gastrointestinal peptide released into the circulation in response to the ingestion of nutrients. GLP‐1 regulates plasma glucose level through stimulation of glucose‐dependent insulin secretion, suppress glucagon secretion, and delays gastric emptying with satiety promotion [1]. Gallbladder (GB) and biliary tract disorders are frequent digestive tract abnormalities among obese patients with T2D. Beside the well-known risk factors such as old age, female gender, and obesity, T2D represents an independent risk factor for cholelithiasis. Interestingly, weight reduction is frequently associated with gallbladder and biliary tract disorders. Owing to the rapid increased prevalence of T2D and obesity, worldwide, and the robust prevailing weight reduction interventions to overcome the obesity-related morbidities, biliary tract diseases especially cholelithiasis represents an overwhelming complication in clinical practice [2]. The use of GLP1-RAs represents a potential risk for gallbladder or biliary tract diseases because GLP-1RAs may inhibit gallbladder motility and delay gallbladder emptying by suppressing secretion of cholecystokinin leading to sludging and stone formation. Moreover, GLP-1RAs induced rapid weight loss may lead to supersaturation of the bile with cholesterol and hence increased incidence of gallstones formation [3, 4]. GLP-1RAs-related upper GI symptoms like dyspepsia, anorexia, vomiting, and colic may be confused with cholelithiasis. Missed cholelithiasis may be complicated with deleterious consequences in patients with T2D. The gallbladder and biliary tract diseases potentials were postulated among patients with T2D using GLP-1RAs. However, the specified relative risk of various biliary disorders among users of different GLP-1RAs is still unclear. We aim to clarify the potential risk of clinically relevant cholelithiasis in between different GLP1-RAs.

## Methods

The current study is a multi-center observational cohort study was conducted simultaneously on December 2022, at 4 centers; Mansoura Specialized Medical Hospital, Mansoura University, Dakahilia, Egypt, Zagazig University Hospitals, Sharkia, Egypt, and Kafrelsheikh University Hospital, Kafrelsheikh, Egypt, and Al Yousif Hospital, Alkhobar, Saudi Arabia. Our study included 308 patients with T2D who used GLP1-RAs continuously, for 12 months and control group treated with conventional anti-diabetic regimens without GLP-1RAs. Participants were subdivided into 4 groups. Group I; the control group and included patients with T2D treated with conventional anti-diabetic therapy not including GLP-1RAs (n = 92). Group II; patients with T2D treated with liraglutide (n = 69). Group III; patients with T2D under treatment with dulaglutide (n = 76). Group IV; patients with T2D who administrated semaglutide (n = 71). We excluded patients with cirrhotic liver, history of gall bladder, biliary disorders, pancreatic diseases, interrupted use of GLP-1RAs throughout the study period, and patients used other drugs with potential effects on the motility of biliary tracts such as cholecystokinin, motilin hormone, cholinomimetic agents like prostigmine, erythromycin, cisapride, cholystyramine, intravenous aminoacids, cholinergic agents like atropine, somatostatin, and progesterone, trimebutine, loperamide and ondansetron. Also, patients with pregnancy, breast feeding, patients suffering from endocrine diseases, autoimmune disorders, chronic debilitating diseases like malignancy, decompensated cardiac, pulmonary, renal, or hepatic diseases were ruled-out. All participants were subjected to detailed clinical history taking, thorough general examination with anthropometric measures (Body weight (BW), Body mass index (BMI), Waist circumference (WC), laboratory testing of complete blood count (CBC), liver function tests; alanine aminotransferase (ALT), aspartate aminotransferase (AST), gamma glutamyl transferase (GGT), serum bilirubin, alkaline phosphatase (ALP), renal function tests, glycated hemoglobin (HBA1c), serum lipase, serum amylase, lipid profile, and abdominal ultrasonography. Cholelithiasis was defined as the hardened deposits of digestive fluid that can form in the gallbladder. Clinically relevant cholelithiasis (Symptomatic cholelithiasis) as symptomatic GB and biliary stones that had influenced the patient’s activity enforcing him to seek medical advice e.g., cholecystitis, biliary colic, fatigue, and refractory dyspepsia; (Symptomatic uncomplicated cholelithiasis) or enforced him to seek interventions e.g., cholecystectomy, acute pancreatitis, obstructive jaundice, or cirrhosis (Symptomatic complicated cholelithiasis). Clinically silent cholelithiasis (Asymptomatic cholelithiasis) was identified as GB stones without clinical manifestations and discovered incidentally while reviewing the patients with the criteria above for the research purposes [5]. The dose of the corresponding GLP-1RA was prescribed according to the manufacturer’s instructions on titration of the dose up to the highest tolerated dose. Liraglutide 1.8 mg daily, weekly injection of dulaglutide 1.5 mg, and semaglutide 1 mg in prefilled pens were used in the corresponding groups of the current study.

### Ethical approval

The study was approved by the Institutional Review Board for Clinical Research Committee (IRB) of Mansoura University with the approval code R.22.10.1897.R1 and with the IRB committee, Al Yousif Hospital, Alkhobar, Saudi Arabia, (AYH IRB: 02/03/2023). All procedures were in accordance with the ethical standards of the institutional research committee and with the 1975 Declaration of Helsinki and its later amendments. Written informed consent was approved by the IRB and signed by all participants before enrollment.

### Sample size

Sample size was calculated by using Power Analysis and Sample Size (PASS) Software (version 15, 2017). NCSS, LLC. Kaysville, Utah, USA. Based on a previous systematic review and meta-analysis by He et al. [6], we hypothesized a medium effect size (W = 0.3) when comparing the effect of Liraglutide, Dulaglutide and Semaglutide vs. control on the occurrence of symptomatic cholelithiasis. A sample size of 200 (50 per group) achieves 96% power to detect an effect size (W) of 0.3000 using a 3 degrees of freedom Chi-Square Test with a significance level (α) of 0.05000. Considering a 20% dropout rate, the sample size will be inflated to 250 (approximately 63 per group) is required.

### Statistical analysis

Data were entered and analyzed using IBM-SPSS software (IBM Corp. Released 2020. IBM SPSS Statistics for Windows, Version 27.0. Armonk, NY: IBM Corp.). Qualitative data were expressed as absolute frequency (N) and percentage (%). Quantitative data were initially tested for normality using Shapiro–Wilk’s test with data being normally distributed if p > 0.050. The presence of significant outliers was tested by inspecting boxplots. Quantitative data were expressed as mean ± standard deviation if normally distributed or median (Q1-Q3) if not. Chi-Square or Fisher’s exact test was used to compare categorical data. Quantitative data between groups were compared by one-way ANOVA if normally distributed or Kruskal–Wallis H-test if not. Pre-post data were analyzed by using one-way ANCOVA and Quade's nonparametric ANCOVA. For any of the used tests, results were considered as statistically significant if p-value ≤ 0.050. Appropriate charts were used to graphically present the results whenever needed.

## Results

Table 1 showed the demographic clinical and laboratory characteristics of participants. Pairwise comparison showed a statistically significant difference in BMI, WC, systolic blood pressure (SBP), AST, GGT, amylase, lipase, VLDL-cholesterol, and serum triglycerides between the four groups. BMI was of the highest values in liraglutide group and of lowest values in the control group (p = 0.024). WC showed the highest measures in the liraglutide and the least values in the control group (p = 0.011). Systolic blood pressure (SBP) was significantly of lowest measures in the control group versus all GLP1-RA groups (p = 0.027). AST and GGT showed significantly higher values in the control group than other groups (p < 0.001) for both. Serum lipase and amylase showed the highest values in dulaglutide group than other groups (p < 0.001 for both). Triglycerides and VLDL-cholesterol were significantly elevated in semaglutide group than other groups (p < 0.001). Age, gender, and duration of DM were of non-significant differences between the study groups. SGLT2 inhibitors use was significantly more prevalent among dulaglutide and semaglutide users than the control group (p < 0.001).

**Table 1:** Demographic, clinical and laboratory characteristics of the study groups

Characteristic	Groups				Test of significance	
	Control N = 92	Liraglutide N = 69	Dulaglutide N = 76	Semaglutide N = 71	χ^2^	p-value
Gender						0.834
Male	61 (66.3%)	41 (59.4%)	49 (64.5%)	46 (64.8%)	0.866	
Female	31 (33.7%)	28 (40.6%)	27 (35.5%)	25 (35.2%)		
					**F**	**p-value**
Age (years)	52.5 ± 7.38	52.4 ± 8.38	52.5 ± 7.01	52.7 ± 7.4	0.012	0.998
DM duration (years)	8.38 ± 3.28	9.26 ± 4.02	9.66 ± 3.75	10 ± 4.94	2.556	0.055
					**H **[3]	**p-value**
Body weight (kg)	97 (85.6–109.6)	99 (89.8–112)	100.6 (90.3–113)	101 (90–109)	4.877	0.181
BMI (kg/m^2^)	34.1 (30.8–37)	36 (32.5–40.4)	35.8 (32.8–38.9)	35.7(32.8–39.6)	9.475	0.024*
Waist circumference (cm)	108 (95.9–116.4)	115 (104–123.5)	114 (105–125.8)	113 (101.5–123)	11.223	0.011*
SBP (mmHg)	145 (135–155)	150 (140–160)	150 (140–160)	150 (140–160)	9.188	0.027*
DBP(mmHg)	90 (80–95)	90 (90–95)	90 (80–100)	90 (90–100)	5.150	0.161
Hemoglobin A1_C_ (%)	9.7 (9.1–10.5)	9.25 (9.3–10.1)	9.75 (9–10.1)	9.79 (9.1–10.1)	0.530	0.912
AST(U/L)	86 (50–85.8)	43 (30–56)	51 (36.5–73.3)	38 (28–57)	48.684	< 0.001*
ALT(U/L)	78.5 (63.3–96)	75 (66–90)	80.5 (62.3–90.8)	74 (66–89)	2.143	0.543
GGT(U/L)	85 (55.5–99)	64 (48–74)	73.5 (48–99.8)	50 (42–70)	28.001	< 0.001*
ALP(U/L)	96.5 (79–113)	100 (90–107.5)	99 (85–110)	98 (86–110)	0.899	0.826
Total bilirubin (mg/dl)	1.06 (1–1.19)	1 (1–1.11)	1.07 (1–1.12)	1.1 (1–1.22)	6.769	0.080
Lipase (U/L)	84.5 (67–98.8)	79 (68–86.5)	93.5 (71.3–108)	92 (71–101)	20.280	< 0.001*
Amylase (U/L)	59.5 (50–69.8)	67 (58.5–75)	70 (60–84.8)	66 (58–71)	32.894	< 0.001*
Platelet count / µl	217 (186–267)	210 (186–269)	247 (199–298)	230 (202–286)	9.432	0.024*
Total cholesterol (mg/dl)	210.5 (189–243)	206 (190–222)	210.5 (190–231)	216 (191–233)	2.751	0.432
HDL-C (mg/dl)	40 (36–46)	43 (34–52)	40.5 (33–51)	40 (34–64)	1.922	0.589
LDL-C (mg/dl)	115.5 (82.5–142)	124 (90–139)	120 (89.3–138.5)	120 (98–142)	1.497	0.683
VLDL-C (mg/dl)	52 (43–65.8)	39 (29–57)	43.5 (33–58.5)	56 (32–72)	18.313	< 0.001*
TG (mg/dl)	265.5 (216–328)	194 (145–284)	216.5 (171–298)	281 (158–320	19.350	< 0.001*
Insulin therapy (N & %)	40 (43.5%)	34 (49.3%)	37 (48.7%)	33 (46.5%)	0.689	.876
Pioglitazone therapy (N & %)	13 (14.1%)	14 (20.3%)	5 (6.6%)	8 (11.3%)	6.310	.097
SGLT-2 inhibitor therapy (N & %)	36 (39.1%) a	34 (49.3%) a, b	49 (64.5%) b	49 (69%) b	18.607	< .001*
Sulfonylureas therapy (N & %)	48 (52.2%)	31 (44.9%)	45 (59.2%)	34 (47.9%)	3.384	.336

Data are N (%) for sex [test of significance is chi-square test], mean ± SD for age & DM duration [test of significance is one-way ANOVA], and median (Q1-Q3) for all other variables [test of significance is Kruskal–Wallis H-test]. * Significant p-value < **0.05**

### Post-treatment changes

The data shown in Table 2 shows the changes reported with the use of GLP1-RAs among T2D patients. Anthropometric indices (BMI, BW, WC) were significantly improved in GLP1-RAs treated patients (p < 0.001), emphasizing the efficacy of GLP-1RAs in weight reduction. Pairwise comparisons revealed that BMI and BW were significantly higher in controls > dulaglutide > liraglutide > semaglutide. Waist circumference (WC), diastolic blood pressure (DBP), AST, ALT, and GGT showed non-significant statistical differences between the three GLP1-RAs groups, but were significantly higher in controls versus all of the three GLP-1RAs groups. Similarly, SBP and parameters of lipid profile including total and differential cholesterols and triglycerides were significantly improved among users of GLP-1RAs. SBP was significantly higher in control > dulaglutide > both liraglutide and semaglutide. Serum total cholesterol, LDL-cholesterol, and serum triglycerides were significantly higher in control group than both semaglutide and liraglutide groups. VLDL-C was significantly lowered in semaglutide than in control cohort.

**Table 2:** Post-treatment clinical and laboratory data adjusted for pre-treatment value as a covariate

Parameter	Control		Liraglutide		Dulaglutide		Semaglutide		F	p-value	Partial η^2^
	Unadjusted	Adjusted	Unadjusted	Adjusted	Unadjusted	Adjusted	Unadjusted	Adjusted			
BMI	35 ± 5.7	36.5 (0.17)	33 ± 5.3	32 (0.19)	33.3 ± 5	32.9 (0.18)	31.5 ± 4.7	31 (0.19)	182.7	< 0.001*	0.660
BW	98 ± 16.6	101.7 (0.39)	94 ± 15.7	91.4 (0.45)	94.5 ± 15.8	93.2 (0.42)	89 ± 13.6	88.2 (0.44)	200.2	< 0.001*	0.665
WC	108.7 ± 16	113.2(0.6)	102.7 ± 13	100.5(0.7)	103.4 ± 13.4	101.4(0.67)	102 ± 12	100.5 (0.7)	91.5	< 0.001*	0.475
SBP	139.9 ± 14	142 (0.72)	130.6 ± 7.9	129.2 (0.83)	132 ± 12.9	133 (0.79)	132.2 ± 10	129.7 (0.82)	60.1	< 0.001*	0.373
DBP	86.8 ± 9.3	87.9 (0.64)	78 ± 6	77.7 (0.74)	79.2 ± 9.4	79.4 (0.7)	78.5 ± 7.7	77.3 (0.73)	54	< 0.001*	0.348
A1c	9.2 ± 1.5	9.1(0.1)	7.7 ± 0.7	7.8(0.1)	8.2 ± 0.9	8.3 (0.1)	8 ± 0.86	8.1 (0.1)	79.5	< 0.001*	0.463
AST	53.2 ± 15.1	46.7 (1.2)	28.7 ± 15.6	32 (1.3)	31.7 ± 17	31.7 (1.2)	28.1 ± 13.6	33.4 (1.3)	35.2	< 0.001*	0.259
*ALT	74.8 ± 88.8	74.6 (5)	36.8 ± 9.9	36.4 (5.8)	39.3 ± 13	38.7 (5.5)	36.2 ± 9.9	37.5 (5.7)	77.6	< 0.001*	0.434
GGT	69.2 ± 28.8	65 (1.8)	33.1 ± 19.7	37 (2)	42 ± 20	40.2 (1.9)	35 ± 19.2	39.6 (2)	49.8	< 0.001*	0.330
ALP	101.7 ± 39	101 (1.9)	114.5 ± 33	113.5 (2.2)	114.8 ± 35.6	115.1 (2.1)	106.2 ± 26	107.8 (2.2)	9.896	< 0.001*	0.089
T. Bilirubin	1.1 ± 0.16	1.1 (0.012)	1.1 ± 0.17	1.1 (0.014)	1.13 ± 0.16	1.14 (0.013)	1.1 ± 0.18	1.08 (0.014)	3.717	0.012*	0.038
*Lipase	97.1 ± 79.8	97 (7.8)	109.3 ± 50	119.5 (9.1)	121.1 ± 71.7	116.3 (8.6)	143.2 ± 116	138.8 (8.9)	33.67	< 0.001*	0.249
Amylase	66.5 ± 14.2	72.8 (2.57)	79.6 ± 24.3	78.3 (2.88)	89.5 ± 34	82.3 (2.83)	87.9 ± 34.8	88.6 (2.83)	6.045	0.001*	0.056
Platelet count	226.1 ± 66	234.3 (2.4)	227 ± 57.5	234.5(2.8)	247.5 ± 56.1	235.2(2.7)	237.4 ± 53	232.8 (2.7)	0.134	0.940	0.001
Total Cholesterol	193 ± 38.1	193.1 (3.79)	169.1 ± 38	171 (4.38)	179.5 ± 38.1	180.7 (4.17)	172 ± 41.2	168.8 (4.34)	7.651	< 0.001*	0.070
HDL-C	43.4 ± 10.4	45 (1.16)	44 ± 13	43 (1.34)	47.1 ± 14.4	47.2 (1.27)	43 ± 12.1	43.2 (1.32)	2.219	0.086	0.022
LDL-C	108 ± 37.2	108.8 (3.72)	93.3 ± 39.4	92.6 (4.29)	94.8 ± 37.2	95.7 (4.09)	92.5 ± 41.1	91.1 (4.23)	4.332	0.005*	0.041
VLDL-C	42.3 ± 23.4	41.2 (1.82)	31.4 ± 18.2	35.3 (2.12)	37.1 ± 24.9	37.8 (2)	36.2 ± 19.4	33 (2.09)	3.315	0.020*	0.032
TG	205 ± 85.6	199.7 (7.18)	147.4 ± 74	159.5 (8.33)	171.8 ± 94.7	173.3 (7.89)	173.2 ± 80	166 (8.18)	5.479	0.001*	0.051

Unadjusted data is mean ± SD and adjusted data is mean (SE). The test of significance is one-way ANCOVA and Quade's nonparametric ANCOVA. * Significant p-value < 0.05

### Diabetes control

In the current study diabetes control was assessed by the glycated hemoglobin (HBA1c) as shown in Table 2. HBA1c was significantly higher in control > dulaglutide and semaglutide > liraglutide, but without significant difference between dulaglutide and semaglutide. Although patients treated with GLP-1RAs showed significantly lowered HBA1c in comparison to control group, yet the overall T2D control looks suboptimal.

### Hepato-biliary-pancreatic biochemical profile

GLP1-RAs use was associated with significantly improved levels of serum hepatocellular enzymes; AST and ALT (p < 0.001), meanwhile markers of cholestasis (ALP, bilirubin) were significantly elevated in comparison to control comparators (p < 0.001). ALP was significantly lowered in controls group than in both liraglutide and dulaglutide groups. Serum total bilirubin was statistically significantly higher in dulaglutide than semaglutide group. On the other hand, the pancreatic enzymes; serum amylase and lipase showed a significant rise among GLP-1RAs users (p ≤ 0.001). Serum amylase was significantly higher in semaglutide versus control group. Serum lipase was significantly lower in control group versus all the three GLP-1RAs groups and was lowered in dulaglutide group versus semaglutide group. All these findings potentiate the assumption that GLP-1RAs use is associated with hepato-biliary-pancreatic biochemical changes (Table 2). However, none of our participants developed acute hepatitis nor acute pancreatitis.

### Cholelithiasis

**Fig. 1:**
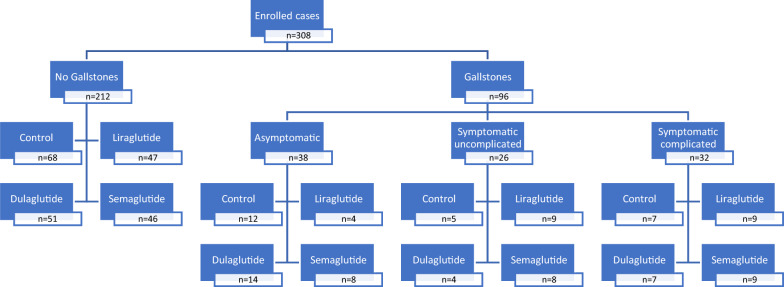
Shows the frequency of various types of clinical relevance of cholelithiasis in the study groups. Out of 308 patients included in the study, cholelithiasis was detected in 96 patients. Asymptomatic cholelithiasis was encountered in 38 patients; 14, 8, 4, and 12 patients with dulaglutide, semaglutide, liraglutide, and non-GLP1-RAs users, respectively. Symptomatic uncomplicated cholelithiasis was noticed in 26 patients; 9, 8, 4, and 5 in liraglutide, semaglutide, dulaglutide, and non-GLP1RAs users, respectively. Symptomatic complicated cholelithiasis was reported in 32 patients; 9, 9, 7, and 7 in liraglutide, semaglutide, dulaglutide, and non-GLP1RAs users, respectively

The frequency of cholelithiasis among the study participants was 31.2% (96/308), and most of the patients with cholelithiasis were symptomatic (58/96; 60.4%); 26 (27.1%) were symptomatic uncomplicated, and 32 (33.3%) were symptomatic complicated meanwhile, 38 (39.6%) were asymptomatic, as shown in Fig. 1. Out of the 38 patients with asymptomatic gallstone (as shown in Fig. 1), 14 (36.8%) were on dulaglutide, 12 (31.6%) were in control group, 8 (21.1%) on semaglutide, and 4 (10.5%) were on liraglutide. Out of the patients with symptomatic uncomplicated gallstone, 9 (34.6%) were on Liraglutide, 8 (30.8%) were on semaglutide, 5 (19.2%) were in control group, and 4 (15.4%) were on dulaglutide. Out of the 32 patients with symptomatic complicated gallstone, 9 (28.1%) were on liraglutide, 9 (28.1%) were on semaglutide, 7 (21.9%) were on dulaglutide, and 7 (21.9%) were not on GLP-1RAs. The highest frequency of symptomatic complicated cholelithiasis was among liraglutide and semaglutide users rather than dulaglutide users. The highest frequency of symptomatic uncomplicated cholelithiasis was among liraglutide users followed by semaglutide with the lowest frequency was among dulaglutide users. On the contrary, the highest frequency of asymptomatic cholelithiasis was among dulaglutide users than other GLP-1RAs users. Table 3 showed the association between GLP-1RAs and symptomatic cholelithiasis. The use of GLP-1RAs was significantly associated with cholelithiasis. Ordinally, symptomatic cholelithiasis was significantly of higher frequency among liraglutide (81.8%) followed by semaglutide (68%) and lastly, dulaglutide (44%) users.

**Table 3:** Association between GLP1 analogues and symptomatic cholecystolithiasis

Gallstone status	Group				Total N = 96	Test of significance		
	Control N = 24	Liraglutide N = 22	Dulaglutide N = 25	Semaglutide N = 25		χ^2^	Cramer’s V	p-value
Asymptomatic	12 (50%) a, b	4 (18.2%) b	14 (56%) a	8 (32%) a, b	38 (39.6%)	8.721	0.301	0.033*
Symptomatic	12 (50%) a, b	18 (81.8%) b	11 (44%) a	17 (68%) a, b	58 (60.4%)			
Risk for cholelithiasis								

Data is N (%). The test of significance is the chi-square test. Cramer’s V is a measure of the strength of association. Multiple z-tests are presented as letters (different letters indicate statistically significant difference). * Significant p-value < 0.05

### The risk of cholelithiasis among different GLP1-RAs

Table 4 showed the overall relative risk of cholelithiasis was 1.2, 1.3, and 1.4 in liraglutide, dulaglutide, and semaglutide versus control with number needed to harm of 17.25, 14.69, and 10.96, respectively. In those with cholelithiasis, the relative risk of symptomatic cholelithiasis was 1.6, 0.9, and 1.4 in liraglutide, dulaglutide, and semaglutide versus control with number needed to harm of 3.14, 16.67, and 5.56, respectively.

**Table 4:** Relative risk for symptomatic and asymptomatic cholelithiasis in GLP1-RAs groups versus control group

Event	Liraglutide			Dulaglutide			Semaglutide		
	RR	NNT (harm)	p-value	RR	NNT (harm)	p-value	RR	NNT (harm)	p-value
Cholelithiasis	1.2	17.25	.419	1.3	14.69	.334	1.4	10.96	.208
Symptomatic cholelithiasis	1.6	3.14	.030*	0.9	16.67	.674	1.4	5.56	.211

RR: relative risk. NNT (harm) = number needed to harm. * Significant p-value < 0.05

## Discussion

In the current study, we found a significant association between GLP-1RAs and cholelithiasis. Overall cholelithiasis was more frequent among GLP-1RAs users than others without GLP1-RAs. In agreement with Dong et al. [7] who conducted a study to detect the risk of biliary-related diseases in patients with T2D using GLP-1RAs versus sodium glucose co-transporters 2 inhibitors (SGLT2I). They reported significantly elevated risk of biliary diseases among GLP-1RAs users with a significant hazard ratio more than 1.2. Additionally, He et al. [6] found a progressively increased risk of gall bladder and biliary disorders with higher doses and longer duration of administration of GLP-1RAs. They noticed seriously increased relative risk of GLP-1RAs-related biliary diseases when used for weight reduction more than for glycemic control; (RR, 2.29; 95% CI 1.64–3.18 versus RR, 1.27; 95% CI 1.14–1.43; P < 0.001). Therefore, the risk of cholelithiasis is postulated to be related to GLP1-RAs rather than T2D.

Our results are consistent with Monami et al. [8] and Nreu et al. [9], who reported higher safety of GLP-1RAs for the risk of acute pancreatitis rather than cholelithiasis in patients with T2D. Furthermore, Faillie et al. [10] noticed doubling the risk of cholecystectomy among patients treated with GLP-1RAs. Hence, the risk of cholelithiasis should be considered and addressed in patients treated with GLP-1RAs [11]. We found significantly increased prevalence of clinically relevant (symptomatic complicated and non-complicated) cholelithiasis among users of GLP-1RAs, in particular, liraglutide and semaglutide. Ordinally, symptomatic cholelithiasis was more frequent among users of liraglutide followed by semaglutide, and finally dulaglutide. Meanwhile, a symptomatic cholelithiasis was more evident in dulaglutide group rather than other GLP-1RAs. In accordance with Dong et al. [7], who noticed higher risk of biliary related diseases in Asian patients who used liraglutide than dulaglutide. In alignment with Nauck et al. [12], who studied liraglutide induced acute biliary adverse events based on data derived from the LEADER trial and reported a significant increased risk of acute biliary complications in patients with liraglutide with (HR: 1.60; 95% CI 1.23, 2.09; *P* < 0.001). Variances in the molecular and pharmacokinetic properties of the aforementioned molecules may explain such differences. The high molecular homology of liraglutide to the native GLP-1(97% amino acid homology) with great protraction properties may explain the high prevalence of symptomatic cholelithiasis among liraglutide users (81.8%) than other GLP-1RAs [13, 14]. In our study, the relative risk of symptomatic cholelithiasis was 1.6, 1.4, and 0.9 in liraglutide, Semaglutide, and dulaglutide versus control group, with the number needed to harm of 3.14, 5.56, and 16.6, respectively. Increased risk of cholelithiasis was explained by Shaddinger et al. [15], and Gether et al. [4], who attributed GLP-1RAs related cholelithiasis to the reduced cholycystokinine mediated gallbladder emptying and prolonged gallbladder refilling that may induce complicated cholelithiasis. On the contrary, Trujillo [16] denied higher incidence of biliary adverse events and acute pancreatitis with administration of GLP-1RAs in patients with T2D. Similarly, Aroda et al., Blonde et al., Jendle et al., [17–19] reported lower incidence of cholelithiasis (0–1%) with GLP-1RAs, nevertheless, they attributed the potential risk of cholelithiasis to weight-loss enhanced lithogenicity. However, variations of the study design, objectives, and population may explain these different views.

GLP-1 receptors are widely expressed in pancreatic islet and exocrine cells. Stimulation of these receptors by GLP-1RAs may lead to cellular overgrowth, hyperplasia, chronic low-grade, or acute inflammation. Fortunately, none of our participants developed acute pancreatitis. We noticed significantly elevated serum lipase, amylase, ALP and GGT without stigma of acute pancreatitis. in consistency with Steinberg et al. [20] who noticed a significant dose dependent reversible rise of pancreatic enzymes; lipase and amylase with liraglutide versus placebo. Storgaard et al. [21], and Li et al. [22] studied the causal relationship between GLP-1RAs and acute pancreatitis and denied significant relationship between GLP-1RAs and acute pancreatitis. Similarly, Cao et al. [23], and Zhang et al. [24] confirmed the high safety profile of GLP-1RAs on the risk of acute pancreatitis and pancreatic cancer. We noticed favorable effects of GLP-1RAs on hepatocellular transaminases with significant reduction of serum levels of ALT, AST, and GGT. Our results agreed with Kuchay et al. [25], and Gameil et al. [26], who reported the beneficial effects of dulaglutide and liraglutide on hepatic aminotransferases in patients with T2D and non-alcoholic fatty liver disease. Improvement of liver enzymes was explained by the direct action of GLP-1RAs on GLP-1 receptors located on the hepatocyte that enhances weight loss, insulin sensitivity, hepatic fat oxidation, reduces denovo lipogenesis, hepatic steatosis, and alleviates oxidative stress [27–34]. Moreover, improved hepatocyte ballooning and steatosis, inhibited inflammatory pathways, mitophagy-mediated active pyroptosis were noticed with liraglutide use in patients with NAFLD [35–37]. In our study, we noticed a significant reduction of LDL-C, TC, VLDL-C, and TG among participants with GLP-1RAs that were in consistency with Aoki et al. [38] and Kahal et al. [39]. On the contrary, Rezaei et al. [40] denied direct effect of GLP-1RAs on the plasma lipid profile. However, they noted various degrees of heterogeneity of studies included in their metanalysis. Ohki et al. [41] explained the liraglutide- related improvement of dyslipidemia by enhanced satiety sensation and weight loss. Our study acquired strength points such as the comparative analysis of 3 different molecules of GLP-1RAs, the real-world applicability of the results that may influence the daily practice in management of T2D. Limitations of the current study included the single ethnicity design, lack of histopathological and molecular assessment tools of GLP-1RAs-mediated cellular changes and lack of sequential invasive tools in assessment of altered gallbladder motility. Moreover, despite the reasonable sample size, the relatively small numbers of patients who experienced various clinical manifestations within the individual subgroups represented additional limitation. Further studies with larger, multi-ethnic diabetic and non-diabetic patients are needed for more relevance of our findings.

## Conclusion

Administration of GLP-1RAs in patients with T2D achieved multiple clinical and metabolic benefits, nevertheless, cholelithiasis was more frequent among patients with GLP-1RAs that should be addressed frequently. Liraglutide followed by semaglutide rather than dulaglutide were associated with relatively higher risk of clinically relevant cholelithiasis.

## Data Availability

No datasets were generated or analysed during the current study.

## Abbreviations

GLP-1RAs: Glucagon like peptide-1 receptor agonists
T2D: Type 2 diabetes mellitus
GB: Gallbladder
GLP‐1: Glucagon‐like peptide-1
ALT: Alanine aminotransferase
AST: Aspartate aminotransferase
GGT: Gamma glutamyl transpeptidase
ALP: Alkaline phosphatase
HBA1c: Glycosylated hemoglobin
NAFLD: Non-alcoholic fatty liver disease
TG: Triglycerides
VLDL-C: Very low-density lipoprotein cholesterol
TG/HDL-C: Triglycerides to high-density lipoprotein cholesterol ratio
LDL-C: Low-density lipoprotein cholesterol
BMI: Body mass index
WC: Waist circumference
WC: Waist circumference
CBC: Complete blood count
SBP: Systolic blood pressure
DBP: Diastolic blood pressure
